# Interleukin 6 plasma levels are associated with progression of coronary plaques

**DOI:** 10.1136/openhrt-2024-002773

**Published:** 2024-09-19

**Authors:** Jordan M Kraaijenhof, Nick S Nurmohamed, Evangelos Tzolos, Mo Meah, Jolien Geers, Yannick Kaiser, Jeffrey Kroon, G Kees Hovingh, Erik S G Stroes, Marc R Dweck

**Affiliations:** 1 Department of Vascular Medicine, Amsterdam University Medical Centres, University of Amsterdam, Amsterdam, The Netherlands; 2 Department of Cardiology, Amsterdam University Medical Centers, Vrije Universiteit Amsterdam, Amsterdam, The Netherlands; 3 British Heart Foundation Centre for Cardiovascular Science, University of Edinburgh, Edinburgh, UK; 4 Department of Cardiology, Universitair Ziekenhuis Brussel, Vrije Universiteit Brussel, Brussels, Belgium; 5 Department of Experimental Vascular Medicine, Amsterdam University Medical Centers, University of Amsterdam, Amsterdam, The Netherlands; 6 Atherosclerosis and Ischemic Syndromes, Amsterdam Cardiovascular Sciences, Amsterdam, The Netherlands; 7 Laboratory of Angiogenesis and Vascular Metabolism, VIB-KU Leuven Center for Cancer Biology, VIB, Leuven, Belgium; 8 Laboratory of Angiogenesis and Vascular Metabolism, Department of Oncology, KU Leuven and Leuven Cancer Institute (LKI), Leuven, Belgium

**Keywords:** Computed Tomography Angiography, Coronary Artery Disease, Inflammation

## Abstract

**Background:**

Inflammation plays a pivotal role in atherogenesis and is a causal risk factor for atherosclerotic cardiovascular disease. Non-invasive coronary CT angiography (CCTA) enables evaluation of coronary plaque phenotype. This study investigates the relationship between a comprehensive panel of inflammatory markers and short-term plaque progression on serial CCTA imaging, hypothesising that inflammation is associated with increased plaque volume.

**Methods:**

A total of 161 patients aged ≥40 years with stable multivessel coronary artery disease were included, who underwent CCTA at baseline and 12 months follow-up. Baseline plasma levels of interleukin 6 (IL-6), high-sensitivity C-reactive protein and other inflammatory markers were measured. Plaque volumes were assessed using semiautomated software, calculating total, noncalcified, calcified and low-attenuation noncalcified plaque volumes. Linear regression models, adjusted for ASSIGN score, segment involvement score and body mass index, evaluated associations between inflammatory markers and plaque volume changes.

**Results:**

The mean±SD age was 65.4±8.4 years, with 129 (80.6%) male participants. Baseline total plaque volume was 1394 (1036, 1993) mm³. After 12 months, total plaque volume changed by 78 (−114, 244) mm³. IL-6 levels were associated with a 4.9% increase in total plaque volume (95% CI: 0.9 to 8.9, p=0.018) and a 4.8% increase in noncalcified plaque volume (95% CI: 0.7 to 8.9, p=0.022). No significant associations were observed for other inflammatory markers.

**Conclusions:**

Plasma IL-6 levels are significantly associated with increased total and noncalcified short-term plaque progression in patients with stable coronary artery disease. This supports the potential of IL-6 as a target for reducing plaque progression and cardiovascular risk.

**WHAT IS ALREADY KNOWN ON THIS TOPIC**

- Inflammation is a risk factor for atherosclerotic cardiovascular disease (ASCVD), but how inflammatory markers are related to coronary CT angiography-derived plaque progression is unknown.

**WHAT THIS STUDY ADDS**

- This study demonstrates that interleukin 6 (IL-6) is significantly associated with the progression of both total and noncalcified plaque burden over a 12 month period in asymptomatic patients with stable coronary artery disease. This finding highlights IL-6 as potential driver of plaque progression, providing new insights into the role of inflammation in ASCVD.

**HOW THIS STUDY MIGHT AFFECT RESEARCH, PRACTICE OR POLICY**

- The findings of this study suggest that targeting IL-6 could be a viable strategy for reducing plaque progression and, consequently, ASCVD risk.

## Introduction

 Non-invasive coronary CT angiography (CCTA) offers a means to evaluate coronary plaque phenotype, and allows the identification of patients at risk of cardiovascular events.^1^ Numerous studies have shown that, in addition to the degree of coronary stenosis, total plaque burden is a strong predictor of future cardiovascular events.^2^,^4^ Studies involving serial imaging have shown that patients with plaque progression are at the highest risk for cardiovascular events. Those who experience such events exhibit an approximately two- to threefold higher rate of plaque progression compared with those who do not experience cardiovascular events.^5 6^

Inflammation is recognised as a pivotal factor in the development of atherosclerosis and has been identified as both an independent and causal risk factor for atherosclerotic cardiovascular disease (ASCVD).^7^ Inflammatory markers have been shown to be associated with adverse plaque characteristics in cross-sectional CCTA studies.^8^ Recent research has demonstrated a correlation between plaque progression and elevated levels of high-sensitivity C-reactive protein (hsCRP),^9^ a widely used but non-causal marker of systemic inflammation. However, whether other atherogenic inflammatory plasma markers are associated with coronary plaque progression remain largely unknown.

In the present exploratory study, we investigated the relationship between a comprehensive panel of inflammatory markers and short-term plaque progression on serial CCTA imaging.

## Methods

### Study design

The current study is an unplanned post hoc analysis of the DIAMOND trial (Dual Antiplatelet Therapy to Inhibit Coronary Atherosclerosis and Myocardial Injury in Patients With Necrotic High-Risk Coronary Plaque Disease). This trial was a double-blind, randomised, parallel-group, placebo-controlled study conducted at a single site in Edinburgh, UK between March 2015 and March 2017.^10^ The study received approval from the local Institutional Review Board, the Scottish Research Ethics Committee (REC reference: 14/SS/0089), the Medicines and Healthcare products Regulatory Agency and the UK Administration of Radiation Substances Advisory Committee. All procedures were carried out in compliance with the Declaration of Helsinki, and written informed consent was obtained from all participants.

### Study population

We enrolled patients aged 40 years or older, with clinically stable multivessel coronary artery disease who underwent CCTA imaging at baseline and after 12 months of follow-up, as described previously.^11^ Multivessel disease was characterised by the presence of significant narrowing (>50% luminal stenosis) or prior revascularisation (either percutaneous coronary intervention or coronary artery bypass graft surgery) in at least two major epicardial vessels. Exclusion criteria for patients included any coronary revascularisation within the last 3 months or an acute coronary syndrome episode within the prior year.

### Coronary imaging

Baseline and follow-up CCTAs were conducted using a hybrid positron emission tomography–CT scanner (64-multidetector Biograph CT, Siemens Medical Systems). Participants with a resting heart rate exceeding 65 beats per min were administered oral β-blockers (50–100 mg of metoprolol) unless contraindicated, prior to scanning. An ECG-gated, breath-held noncontrast CT scan (tube voltage 120 kV; tube current adjusted to body habits) was performed for coronary calcium scoring and reconstructed in the axial plane with a 3 mm slice thickness and 1.5 mm increment. Subsequently, an ECG-gated coronary CT angiogram (tube voltage 120 kV, tube current adjusted to body habitus) was acquired in mid-diastole during a held expiration following the administration of sublingual glyceryl trinitrate.

CCTA segments and vessels were identified using anatomical landmarks such as bifurcations and side branches. Segments and vessels containing stents were excluded from the analyses, ensuring that an equal number of segments and vessels were evaluated at both baseline and follow-up. A segment-wise analysis was conducted according to the 17-segment modified American Heart Association classification.^12^ CCTA images were analysed by a trained observer who was blinded to the patient’s clinical status,^13^ ensuring high reproducibility,^14^ using semiautomated software (AutoPlaque V.2.5, Cedars-Sinai Medical Center).^11^ Coronary artery centrelines were extracted in a semiautomated fashion for each major artery and any tributary of >2 mm diameter with visually observed disease. A region of interest was placed in the aorta to define blood pool attenuation. Coronary artery segments were defined manually according to Society of Cardiovascular CT guidance.^15^ Vessels that were revascularised were excluded, allowing for a one-to-one comparison of baseline and follow-up-plaque volumes. A segment involvement score was determined by counting all coronary artery segments that contained plaque, regardless of the extent of luminal stenosis observed in each segment (range 0–16).^16^

### Laboratory assessments

Baseline plasma and serum samples were collected at the time of enrolment and preserved at −80°C for future analysis. The plasma levels of interleukin 6 (IL-6), tumour necrosis factor alpha (TNFα), interferon gamma (INγ), interleukin 8 (IL-8), monocyte chemoattractant protein-1 (MCP-1), vascular cell adhesion molecule 1 (VCAM-1), intercellular adhesion molecule 1 (ICAM-1) and serum amyloid A (SAA) were measured in duplicate using an electrochemiluminescent (ECLIA, Meso Scale Discovery) assay, while interleukin 18 (IL-18) and myeloperoxidase (MPO) were measured using a bead-based immunoassay multiplex (Luminex, R&D systems) assay. Plasma hsCRP levels were measured on a Cobas c702 analyzer (Roche Diagnostics, Mannheim, Germany).

### Statistical analysis

The plasma markers were normalised using Z-score standardisation to facilitate comparison. The percentage change in plaque volume was calculated by dividing the difference between follow-up and baseline plaque volumes by the baseline volume, multiplied by 100%. The relationship between the inflammatory markers and plaque volume was assessed using linear regression models, adjusted for the ASSIGN score (a Scottish cardiovascular risk score incorporating the cardiovascular risk factors age, sex, smoking, blood pressure, total and high-density lipoprotein cholesterol, diabetes, rheumatoid arthritis and deprivation index),^17^ segment involvement score and body mass index (BMI). Plaque volumes and plasma inflammatory marker levels are presented as median with IQR. The significance level was set at a p-value below 0.05 in two-sided statistical analyses, conducted using RStudio V.4.3.2 (R Foundation, Vienna, Austria).

## Results

A total of 161 patients underwent serial imaging and had baseline blood sampling available.^11^ The mean±SD age was 65.4±8.4 years, 129 (80.6%) were male and a total of 153 (95.6%) were using statins (table 1). Plasma levels of IL-6 and hsCRP were 1.2 [0.9, 1.7] pg/mL and 1.2 [0.6, 2.0] mg/L, respectively. The other baseline plasma parameters are presented in table 1. The baseline total plaque volume was 1394 [1036, 1993] mm^3^, noncalcified plaque volume was 1280 [955, 1683] mm^3^, calcified plaque volume was 99 [37, 226] mm^3^ and low-attenuation noncalcified plaque volume was 88 [51, 168] mm^3^. After 12 months, total plaque volume changed by 78 [−114, 244] mm³, noncalcified plaque volume changed by 76 (−93, 227) mm³, calcified plaque volume changed by 3 [−13, 35] mm³ and low-attenuation noncalcified plaque volume changed by 1 [−23, 26] mm³.

**Table 1:** Baseline characteristics

Characteristics	Cohort, n=161
Age (years)	65.4 (8.4)
Male sex (%)	129 (80.6)
Current smoker (%)	21 (12.8)
Diabetes mellitus (%)	28 (17.1)
BMI (kg/m^2^)	29.4 (4.9)
Systolic blood pressure (mm Hg)	146.8 (19.2)
Diastolic blood pressure (mm Hg)	81.2 (10.2)
Creatinin (µmol/L)	80.1 (12.7)
Statin use (%)	153 (95.6)
Total cholesterol (mmol/L)	4.2 (1.0)
LDL cholesterol (mmol/L)	2.2 (0.8)
HDL cholesterol (mmol/L)	1.2 (0.3)
Triglycerides (mmol/L)	1.5 [1.1, 2.2]
Lipoprotein (a) (mmol/L)	14.4 [5.6, 58.5]
Interleukin 6 (pg/mL)	1.2 [0.9, –1.7]
Interleukin 18 (pg/mL)	94.7 [67.7, 126.4]
TNFα (nmol/L)	1.5 [1.2, 1.9]
INγ (nmol/L)	6.7 [4.7, 11.1]
Interleukin 8 (pg/mL)	5.9 [4.6, 7.2]
MCP-1 (pg/mL)	152 [124, 174]
VCAM-1 (ng/mL)	508 [431, 603]
ICAM-1 (ng/mL)	491 [422, 639]
SAA (mg/L)	2.0 [1.2, 3.3]
MPO (pg/mL)	234 [189, 293]
hsCRP (mg/L)	1.2 [0.6, 2.0]

Baseline characteristics. Continuous variables with a normal distribution are reported as mean (SD). Continuous variables with a non-normal distribution are reported as median ± IQR.

BMIbody mass indexHDLhigh-density lipoproteinhsCRPhigh-sensitivity C-reactive proteinICAM-1intercellular adhesion molecule-1INγinterferon gammaLDLlow-density lipoproteinMCP-1monocyte chemoattractant protein 1MPOmyeloperoxidaseSAAserum amyloid ATNFαtumour necrosis factor alphaVCAM-1vascular cell adhesion molecule-1

IL-6 levels were associated with change in total plaque volume after 12 months. Every SD increase of IL-6 was associated with a 4.9% increase in total plaque volume (95% CI: 0.9 to 8.9, p=0.018) as well as a 4.8% increase in noncalcified plaque volume (95% CI: 0.7 to 8.9, p=0.022; table 2). IL-6 was not associated with calcified plaque progression or low-density noncalcified plaque progression. The other inflammatory markers measured were not associated with change in total plaque volume or other plaque volumes (table 2).

**Table 2:** Association between plasma inflammatory markers and CCTA progression

	Total plaque volume		Calcified plaque volume		Noncalcified plaque volume		Low-density noncalcified plaque volume	
	β (95% CI)	P value	β (95% CI)	P value	β (95% CI)	P value	β (95% CI)	P value
IL-6	4.87 (0.85 to 8.89)	**0.018**	2.25 (−14.18 to 18.67)	0.787	4.81 (0.71 to 8.91)	**0.022**	4.62 (−5.36 to 14.59)	0.361
IL-18	−0.62 (−4.40 to 3.17)	0.747	−0.42 (−9.40 to 8.56)	0.926	−0.45 (−4.31 to 3.41)	0.817	4.12 (−5.27 to 13.52)	0.387
TNFα	−0.98 (−5.99 to 4.03)	0.699	−7.41 (−19.47 to 4.66)	0.227	−0.71 (−5.82 to 4.40)	0.784	−6.22 (−18.65 to 6.22)	0.324
INγ	2.08 (−2.94 to 7.11)	0.414	7.49 (−4.4 to 19.37)	0.215	2.13 (−2.99 to 7.26)	0.411	−0.63 (−13.1 to 11.83)	0.920
IL-8	1.02 (−3.18 to 5.23)	0.631	5.56 (−4.87 to 15.99)	0.294	0.47 (−3.82 to 4.76)	0.828	−6.4 (−16.83 to 4.03)	0.227
MCP-1	−1.23 (−4.75 to 2.30)	0.493	−0.91 (−9.30 to 7.48)	0.830	−1.17 (−4.77 to 2.43)	0.521	−5.64 (−14.40 to 3.11)	0.204
VCAM-1	−3.31 (−7.82 to 1.20)	0.149	−6.66 (−17.46 to 4.14)	0.224	−3.02 (−7.63 to 1.58)	0.196	3.24 (−7.96 to 14.43)	0.568
ICAM-1	−0.1 (−4.02 to 3.82)	0.960	−2.24 (−11.50 to 7.02)	0.633	0.37 (−3.63 to 4.36)	0.856	−1.06 (−10.78 to 8.66)	0.829
SAA	−1.72 (−5.57 to 2.12)	0.376	−6.98 (−16.19 to 2.24)	0.136	−1.58 (−5.50 to 2.34)	0.426	−1.46 (−11.00 to 8.07)	0.762
MPO	0.74 (−2.96 to 4.43)	0.694	3.56 (−5.32 to 12.43)	0.429	0.07 (−3.69 to 3.84)	0.970	0.65 (−8.51 to 9.81)	0.889
hsCRP	−2.5 (−6.85 to 1.85)	0.257	4.86 (−7.37 to 17.1)	0.433	−3.03 (−7.47 to 1.41)	0.179	−3.42 (−14.22 to 7.37)	0.532

Results of the regression analyses with the β (beta) coefficients presented with 95% CI for the absolute change in plaque volume (mm3) for the following markers

The multivariable linear regression analysis was adjusted for the ASSIGN and segment involvement score and Body Mass Index.

CCTAcoronary CT angiographyhsCRPhigh-sensitivity C-reactive proteinICAM-1intercellular adhesion molecule-1IL-6interleukin 6IL-8interleukin 8IL-18interleukin 18INγinterferon gammaMCP-1monocyte chemoattractant protein 1MPOmyeloperoxidaseSAAserum amyloid ATNFαtumour necrosis factor alphaVCAM-1vascular cell adhesion molecule-1

## Discussion

**Figure 1:**
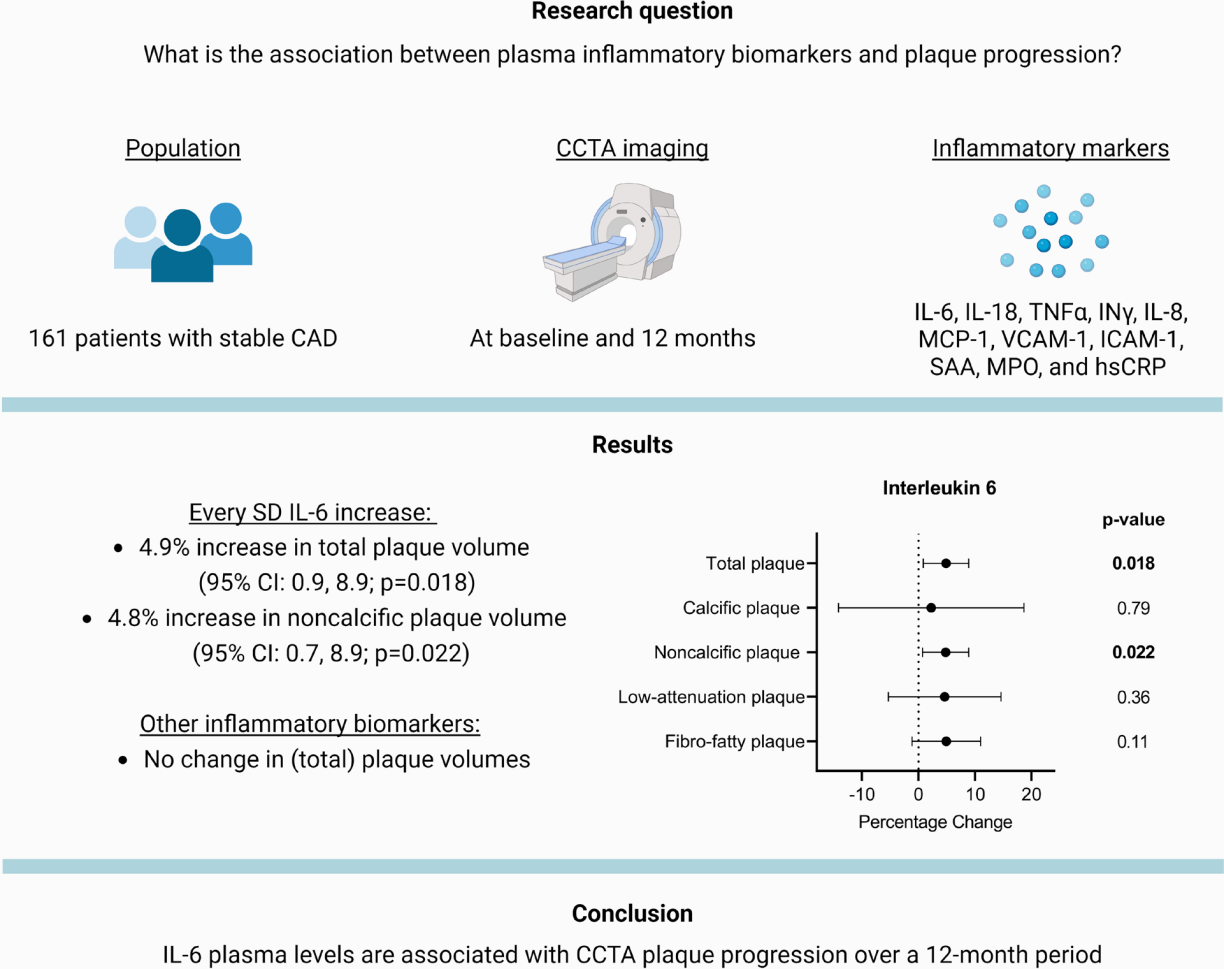
Association of plasma inflammatory markers IL-6, IL-18, TNFα, INγ, IL-8, MCP-1, VCAM-1, ICAM-1, SAA, MPO and hsCRP with percentage change in CCTA plaque progression over a 12 month period. Results from regression analyses show that each SD increase in IL-6 levels was associated with a 4.9% increase in total plaque volume (95% CI: 0.9 to 8.9, p=0.018) and a 4.8% increase in noncalcified plaque volume (95% CI: 0.7 to 8.9, p=0.022). Other inflammatory markers did not show a significant association with plaque progression. The models were adjusted for factors included in the ASSIGN score, which encompasses multiple cardiovascular risk factors such as age, sex and smoking, along with the segment involvement score and Body Mass Index. The study comprised 161 patients with serial imaging data, with a mean±SD age of 65.4±8.4 years, 152 (80%) of whom were male and 153 (95.6%) were on statin therapy. The baseline total plaque volume was 1394 [1036–1993] mm^3^, noncalcified plaque volume was 1280 [955–1683] mm^3^, calcified plaque volume was 99 [37–226] mm^3^ and low-attenuation plaque volume was 88 [51–168] mm^3^. Created with Biorender.com. CAD, coronary artery disease; CCTA, coronary CT angiography; IL-6, interleukin 6; IL-18, interleukin 18; TNFα, tumour necrosis factor alpha; INγ, interferon gamma; IL-8, interleukin 8; MCP-1, monocyte chemoattractant protein-1; VCAM-1, vascular cell adhesion molecule 1; ICAM-1, intercellular adhesion molecule 1; SAA, serum amyloid A; MPO, myeloperoxidase; hsCRP, high-sensitivity C-reactive protein.

Here, we show that IL-6 is associated with increased progression of both total and noncalcified plaque burden among asymptomatic patients with stable coronary artery disease (figure 1). IL-6 is a unique proinflammatory cytokine within the NLRP3 inflammasome pathway and has been identified as a key player throughout the atherogenic process. Its effects include promoting endothelial cell activation and increasing vascular permeability, which facilitate plaque formation through the influx of immune cells and lipoproteins. Within the vessel wall, IL-6 may further stimulate plaque progression by activating macrophages and stimulating vascular smooth muscle proliferation. Murine models have shown that IL-6 instigate both an accelerated formation and a destabilisation of atherosclerotic plaques.^18^

In assessing plaque phenotype through CCTA, noncalcified, soft/low-attenuation, plaques are particularly susceptible to destabilisation^2^ and have been independently associated with cardiovascular events.^3 19^ In the PARADIGM (Progression of Atherosclerotic Plaque Determined by Computed Tomographic Angiography Imaging) registry which included over 1,000 patients with serial CCTAs, progression of the noncalcified plaque component, besides total plaque burden, showed the strongest association with cardiovacsular events.^20^ The association of elevated IL-6 levels, with both total and noncalcified plaque progression as observed in our study, supports the theory that systemic inflammation accelerates the development of coronary artery plaques and may confirm that such inflammation contributes to the formation of a more vulnerable, noncalcified plaque type.

Other inflammatory plasma markers in our study were not associated with changes in plaque volume. These markers of vascular injury (VCAM-1, ICAM-1 and SAA), proinflammatory signalling (TNFα, INγ and IL-18) and chemotactic activity (MCP-1 and IL-8) are well recognised for their roles in plaque formation, as extensively studied in experimental research.^21 22^ The lack of association in our study could be attributed to the complex roles that these markers play in stimulating plaque formation, unlike IL-6, which influences all stages of atherogenesis.^21^ For instance, chemokines involve numerous receptors that can interact with a variety of ligands, and both ligands and receptors may also form heteromers, making the chemokine ligand-receptor network complex.^23^ Furthermore, as chemokine expression varies between advanced atherosclerotic plaques and early-stage lesions,^24^ it could be challenging to link these markers at a single point in time with coronary plaque progression.

Major limitations are the small study population and the short duration of follow-up, which may have impacted the ability to detect changes in the less abundant plaque types. The inclusion of secondary prevention patients, along with a predominantly male population and frequent statin use, might limit the generalisability of our findings. The absence of an association with hsCRP, as opposed to the findings of Bienstock *et al*,^9^ may be attributed to the smaller sample size and the lower and less variable hsCRP levels in our study, but might also be explained by hsCRP’s non-causal association with ASCVD and its weaker association with clinical events compared with IL-6, suggesting that IL-6 might hold greater clinical relevance as a risk factor for ASCVD compared with the relatively nonspecific hsCRP.^25^

In conclusion, the present exploratory serial CCTA imaging study reveals a significant association between IL-6 levels and plaque progression over a 12 month period in statin-treated patients with ASCVD. These data suggest that anti-inflammatory strategies, particularly IL-6 inhibition, could potentially contribute to reducing plaque progression and ASCVD risk.

## Data Availability

Data are available upon reasonable request.
